# Mirror-Mark Tests Performed on Jackdaws Reveal Potential Methodological Problems in the Use of Stickers in Avian Mark-Test Studies

**DOI:** 10.1371/journal.pone.0086193

**Published:** 2014-01-27

**Authors:** Manuel Soler, Tomás Pérez-Contreras, Juan Manuel Peralta-Sánchez

**Affiliations:** 1 Departamento de Zoología, Facultad de Ciencias, Universidad de Granada, Granada, Spain; 2 Grupo Coevolución, Unidad Asociada al CSIC, Universidad de Granada, Spain; 3 Departamento de Microbiología, Facultad de Ciencias, Universidad de Granada, Granada, Spain; 4 Knight Lab, Jennie Smoly Caruthers Biotechnology Building, Boulder, Colorado, United States of America; CNR, Italy

## Abstract

Some animals are capable of recognizing themselves in a mirror, which is considered to be demonstrated by passing the mark test. Mirror self-recognition capacity has been found in just a few mammals having very large brains and only in one bird, the magpie (*Pica pica*). The results obtained in magpies have enormous biological and cognitive implications because the fact that magpies were able to pass the mark test meant that this species is at the same cognitive level with great apes, that mirror self-recognition has evolved independently in the magpie and great apes (which diverged 300 million years ago), and that the neocortex (which is not present in the bird's brains) is not a prerequisite for mirror self-recognition as previously believed. Here, we have replicated the experimental design used on magpies to determine whether jackdaws (*Corvus monedula*) are also capable of mirror self-recognition by passing the mark test. We found that our nine jackdaws showed a very high interest towards the mirror and exhibited self-contingent behavior as soon as mirrors were introduced. However, jackdaws were not able to pass the mark test: both sticker-directed actions and sticker removal were performed with a similar frequency in both the cardboard (control) and the mirror conditions. We conclude that our jackdaws' behaviour raises non-trivial questions about the methodology used in the avian mark test. Our study suggests that the use of self-adhesive stickers on sensitive throat feathers may open the way to artefactual results because birds might perceive the stickers tactilely.

## Introduction

Charles Darwin [1] asserted that there is continuity between human mental capabilities and those of other animals. During the last three decades a great deal of research has been made on the biological bases of cognition and it has been found that many characteristics that had been presumed to be unique to humans may also be found in other animals [2]–[3].

More recently, it has also been pointed out that, contrary to traditional beliefs, primates do not constitute the pinnacle of cognition capacity. Corvids, a group of birds which belong to the order of Passeriformes and which are known to have allometrically large brains [4], have been found to possess at least a similar repertoire of complex cognitive abilities as those of primates [5]. For example, a great capacity for solving novel problems [6], an episodic-like memory [7], ceremonial-like gatherings in response to dead conspecifics [8]–[9], and other sophisticated cognitive functions [10]–[13]. Furthermore, corvids are similar to primates in some highly complex cognitive abilities [5]. For instance, several species are able to hide thousands of seeds and are capable of remembering where and when they were cached [14]; in addition, some species are capable of bearing in mind whether or not they were observed by other birds when concealing food [15]. Siberian jays (*Perisoreus infaustus*) provide information to conspecifics, not only about predator identity, but also about predator behaviour [16]–[17]. American crows (*Corvus brachyrhynchos*) are capable of recognizing individual humans and remembering who are dangerous and who provided any kind of aid in the past [18]–[19]. It has been experimentally demonstrated that western scrub-jays (*Aphelocoma californica*), are able to store food according to exact forecasts of future needs [20]. With respect to tool use and tool manufacture, New Caledonian crows (*Corvus moneduloides*) also do better than primates as one individual was capable of manufacturing tools according to her needs [21].

Self-awareness is a cognitive function typical of humans that is achieved by children when they are between 18 and 24 months of age [22]–[23], and it has been interpreted that animals that pass the mark test are capable of recognizing themselves in a mirror [24]–[25]. This means that at least some individuals of those species are capable of identifying their own reflection in a mirror and, if experimentally marked with a visible coloured mark on their face (which is only visible in the mirror), they use their mirror-image to touch that mark [26]. The conclusion that self-directed behaviour in response to a mirror implies some form of human-like self-awareness is not free from controversy [27]–[29]; however, to some, it is clear that passing the mark test implies capacity for mirror self-recognition [30].

Mirror-induced self-directed behaviour has been studied in many species. Most of them fail to show self-directed behaviour in front of their mirror-image, but many respond to the self-image with social behaviour, i.e. treating the mirror-image as if it were a conspecific. This is also the case for most avian species [31]–[34]. Some species are capable of solving other tasks that require more sophisticated cognition abilities such as discriminating among different objects or using the mirror-image to locate hidden food, which has also been found in a few avian species [31], [34]. However, mirror self-recognition has been found in just a few mammals having very large brains [26], [35]–[36] and in only one bird, the magpie (*Pica pica*) [37].

Prior et al. [37], in a carefully designed and well-controlled experiment, found that magpies confronted with their mirror-image at the beginning responded with social behaviour (aggressive and submissive displays) and exploration of the mirror (approaching it and looking behind it), but later showed self-contingent behaviour (i.e. rapid left and right or back and forth movements in front of the mirror). After 250 min of cumulative exposure to the mirror, each magpie was subjected to eight sessions of the mark test, twice on each of four different conditions (as described in Prior et al. [37]). In four of the sessions they were provided with a brightly coloured sticker and in the other four with a black (sham) sticker. Similarly, four of the sessions were performed with a mirror in the cage, and the other four with a non-reflective plate of the same size in the cage. Two out of five magpies were reported to pass the mark test. The sticker was stuck under the beak, in the throat region, outside the magpies' visual field, but these two magpies were capable of removing the sticker by scratching with their foot in mirror-present sessions. When the magpies were tested with the non-reflective plate, evidence of sticker-directed behaviour was negligible [37].

The results published by Prior et al. [37] proved fascinating because they implied that human-like mirror self-recognition has evolved in a bird species, which challenged the fact that this capacity had previously been found in only a few species of large-brained mammals. This was especially intriguing taking into account that there is a great phylogenetic distance between mammals and birds, which implies substantial differences in the anatomic organization of their forebrains [38]; mainly, the fact that birds' telencephalon lacks the laminated cortex typical of the mammal brain [39]. Findings by Prior et al. [37] imply that similar selection pressures for complex cognitive abilities in mammals and birds have driven convergent evolution of cognitive skills in both vertebrate classes [38].

This study replicates the experimental design used by Prior et al. [37] on magpies with the aim of determining whether jackdaws (*Corvus monedula*) are also capable of passing the mark test. The jackdaw is a good candidate for mirror self-recognition because it is also a food-storing corvid species [40], as corvids in general has an allometrically large brain [4]–[5], [41], sophisticated cognitive abilities [42], and a complex social behaviour [42]–[43]. Furthermore, a recent experimental study has shown that another *Corvus* species, the New Caledonian crow, has an ability of processing mirror information comparable to that shown by non-human primates and children [34].

## Materials and Methods

### Ethics Statement

Research was conducted according to national (Real Decreto 1201/2005, de 10 de Octubre) and regional guidelines. The study was approved by the Ethics Committee of the University of Granada (Comité de Ética en Experimentación Animal, CEEA, Ref.: 785). All necessary permits, including that for confinement of jackdaws, were obtained from the Consejería de Medio Ambiente de la Junta de Andalucía, Spain. The study did not involve endangered or protected species. All efforts were made to minimize suffering and no bird showed symptoms of stress or died during this study.

### Study subjects, housing and experimental conditions

This study was performed with nine adult jackdaws, which were maintained in an outdoor aviary of approximately 240 m^3^, located in the Hoya de Guadix (southern Spain, a high-altitude plateau, approx. 1000 m a.s.l., near Hernán Valle, 60 km from Granada). Six jackdaws were two years old and were hand-raised; the other three, captured in the wild, were of unknown age. The six hand-raised jackdaws were collected when they were nestlings about 12 days old, from four different nests located in the Hoya de Guadix. The three wild jackdaws were also captured in a cage-trap placed very close to the aviary, as wild jackdaws frequently “visit” the captive jackdaws. All of them (four males and five females) bred in captivity during the 2011 breeding season. These birds had not participated in any other experiment in the past. All jackdaws were marked with a unique combination of coloured leg bands for individual identification. Investigation of behaviour towards the mirror with jackdaws was performed between 26 January and 11 February 2012.

The birds were provided with bread, cracked grains of wheat and rice, apple, lettuce, fodder for dog puppies and water ad libitum. We also provided jackdaws with minced meat mixed with food manufactured for canaries with honey and small pieces of fruit (eggfood with fruits, Bogena) and, during the breeding season, boiled eggs and fly maggots. The jackdaws' wellbeing was followed by monitoring their physical condition when providing the food (every two-three days or daily during the breeding season), and once per year, in January, all the birds were captured, measured and examined in details.

The aviary in which the nine adult jackdaws together with the juveniles reared during the 2011 breeding season were maintained consisted of four cages of about 50 m^3^ each, interconnected by holes of about 25 cm in diameter through a central cage (of about 40 m^3^) in which food was provided. Two weeks before the experimental sessions the nine adult jackdaws were isolated in one of the cages.

**Figure 1:**
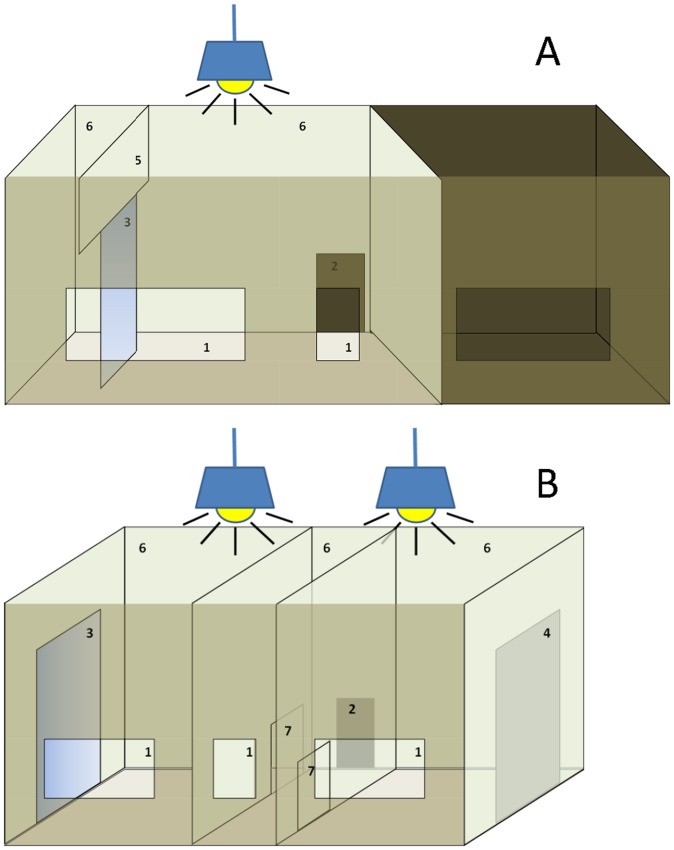
Diagrams of the box used in the experimental sessions. (A) The non-compartmentalized experimental box, (B) the compartmentalized experimental box (see text for a detailed description). Specified numbers indicate: (1) observation windows, (2) entrance to the experimental box, (3) mirror, (4) cardboard, (5) piece of agglomerate placed on the top of the mirror (or the cardboard) to prevent jackdaws from perching on it, (6) the roof of the box, which was of methacrylate to allow clear illumination into the box, and (7) entrances to both compartments.

The experimental sessions were conducted in a box made of agglomerate board (160×100×80 cm; length×height×width) that could easily be adapted to the necessities of each experimental test by making it smaller (fourth and fifth experimental stages; see Figure 1A) or separating it with two sheets of agglomerate board into two identical compartments of 70×100×80 cm, divided by a corridor of 20 cm width (third experimental stage; see Figure 1B). In this case, the entrance to one compartment was closer than the entrance of the other and the bird was released in such way that one entrance was invariably closer than the other entrance (see Video S1 in supplementary material). No perches were provided in the experimental box, forcing the experimental subjects to stay on the ground at the same level as the mirror. One window of 20×30 cm allowed direct observation in each compartment and another one of 20×10 cm observation of the corridor. The windows were covered by smoked glass, which enabled watching and filming without the birds seeing anything outside the box. Thus, in this experimental box birds were forced to concentrate on the experimental situation. The birds could not see their reflection in the windows because these were higher than their eyes. The mirror, 50×60 cm (width×height), thoroughly cleaned before each session, was placed directly on the ground. We placed the mirror vertically because vertical mirrors have been proven to be more effective in eliciting ‘mirror-image’-directed responses than horizontal mirrors [31], [32].

The roof of the box was of methacrylate, which allowed clear illumination into the box by installing two lamps on the roof. The box was set in a windowless experimental room located in the laboratory, 50 m from the aviaries.

Each bird was captured in its cage, placed in a bag and taken to the laboratory. The light in the room was turned off and the lights illuminating the box located on the methacrylate were turned on just before the bird was released into the experimental box. Then, the bird was released in the box, at the entrance of the corridor, directly in front of the observation window at the other side of the box (see Figure 1B).

### Experimental design

We followed almost exactly the experimental protocol described by Prior et al. [37]; the main difference was that in some cases we diminished the number of sessions in order to decrease the potential stress for each bird. However, the total time of exposure in our study was identical or slightly longer. Our experimental protocol, like that of Prior et al. [37], consisted of five successive experimental stages:

In stage 1, each bird was exposed for 30 min to a baseline session in which a mirror-sized grey, non-reflective cardboard was set in the large experimental box (Figure 1A). The cardboard was set 50 cm away from the wall, in order to allow subjects to move freely around it.

In stage 2, each bird was exposed to the vertical mirror in three subsequent daily sessions of 50 min each in the large experimental box, in which the mirror was set 50 cm away from the wall (as described above; Figure 1A).

In stage 3, in order to quantify mirror-induced behaviour and preference for the mirror, each subject was exposed to the compartmentalized experimental box (see above) in six inconsecutive, 20-min sessions on two different days. Each bird received three 20-min sessions per day. Birds received each next 20-min session only after all the other subjects had been tested (i.e. about 3 h after the previous 20-min session). On every session, each subject was released in the corridor between compartments and could move freely from one to the other. The position of the mirror and the cardboard was switched from one compartment to the other on the second day (see Figure 1B).

In stage 4, to estimate self-directed behaviour we gave each marked individual a total of eight mark-test sessions in the large experimental box (Figure 1A), but with the mirror or the cardboard placed on the wall (as in Figure 1B). These sessions included all four conditions: (1) mirror and coloured (yellow or red) sticker, (2) mirror and black sticker, (3) cardboard and coloured (yellow or red) sticker, and (4) cardboard with black sticker (see in p. 1648, [37] for a detailed discussion of the advantages of this experimental approach). Each session lasted 20 min and each condition was replicated once. We allowed at least 3 h between two consecutive sessions and we randomized conditions and the order of presentation.

**Figure 2:**
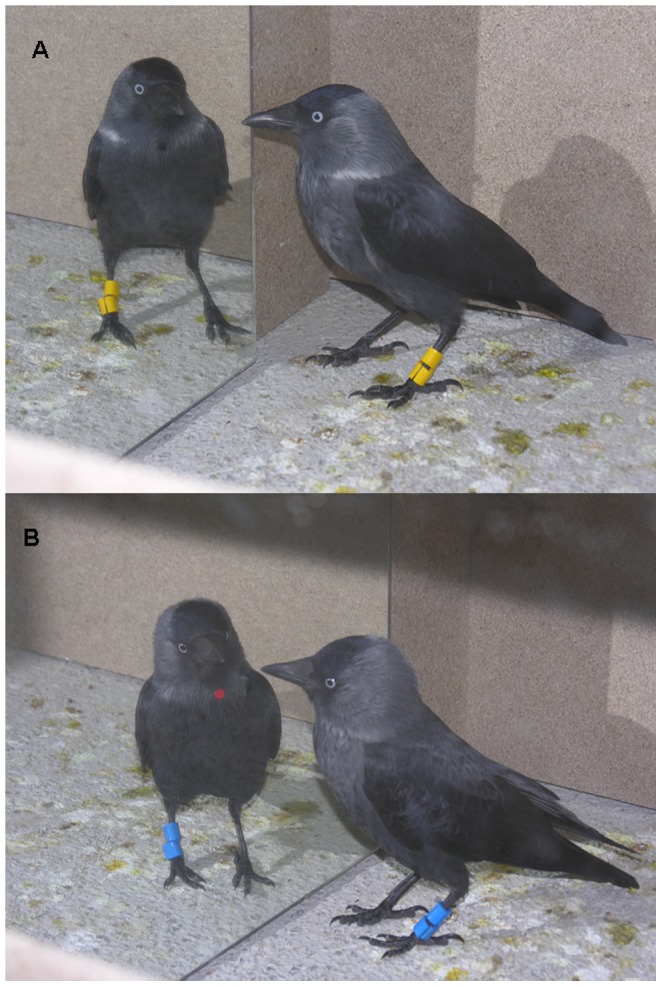
Jackdaws in front of the mirror. (A) With a black mark, (B) with a red mark.

Our stickers were smaller (diameter = 6 mm *vs.* 8 mm) than those used by Prior et al. [37], but much heavier (16 µg in Prior et al. [37] *vs.* 2375±129 µg [average ± standard deviation] in our study). The weight reported by Prior et al. [37] is surprisingly low for a self-adhesive sticker, but, in any case, in the magpie study, the adhesive sticker was fixed using double-sided adhesive tape, the weight of which is not specified in their paper. In our study on jackdaws, we fixed the self-adhesive stickers onto the throat region (Figure 2) without using further adhesive elements, while keeping the bird's head inside the bag. Thus, the bird was prevented from seeing anything during the fixing procedure. The sticker fixed onto the throat region is out of the birds' visual field [38]. The action of pressing the sticker on the throat feathers was repeated 3–5 times on the breast and the wing. These experimental sessions were carried out using only one of the compartments (including the corridor) of our experimental box (see Figure 1A). A comparison between Figure 1 in Prior et al. [37] and our Figure 2 could suggest that the sticker was fixed closer to the base of the beak in the magpie study, but as can be seen in Video S4 and Video S8 in Supplementary Material, the position of the stickers in our jackdaws was usually closer to the base of the beak than in Figure 2. In the magpie study the sticker was sometimes placed beyond the beak (see Video S1, scene 1 and Video S5, scene 1 of Supplementary Material in Prior et al. [37]) or even turned aside towards the right (see Video S1, scene 4 of Supplementary Material in Prior et al. [37]).

Finally, in stage 5 we performed an additional mirror test with Blue-Blue, the jackdaw that showed the highest frequency of close inspections of the mirror and cardboard, the highest frequency of pecks at both the mirror and cardboard, and the highest frequency of self-contingency behaviour in front of the mirror. In this final stage, we gave Blue-Blue four consecutive 5-min mark tests. We changed the colour (i.e. yellow, red, blue and black) of the sticker after each test.

### Scoring of behaviour

In all experimental sessions the behaviour of the birds was directly observed through the smoked glass windows and videotaped (using a Panasonic HDC-SD40 camera). Stages 1 and 2 were captured on video from a hole in front of the mirror (as shown in Video S2). In stages 3, 4 and 5, experimental sessions were recorded from the smoked glass windows (as shown in Video S3). We scored the birds' behaviour in a similar way to that of Prior et al. [37]. We quantified: (1) time with view of the mirror or the cardboard; (2) time of close inspection of mirror or cardboard (i.e. subject stared directly to the mirror or cardboard from a close range (see Video S4 in supplementary material)); (3) frequency of pecks directed to the mirror or cardboard; (4) frequency of looks behind the mirror or cardboard; (5) frequency of self-contingent behaviour (see below); (6) frequency of social behaviour (i.e. agonistic or submissive displays); (7) frequency of mark-directed behaviour (specifically, directed towards the throat region); and (8) frequency of self-directed behaviour (directed towards stickerless body parts). Following Prior et al. [37], we have considered the bird's jumping and/or flying towards the mirror as social behaviour; however, we think that such movement is, at least sometimes, only an attempt to cross the plane of the mirror.

The behaviour was considered an indication of self-contingency when the subject moved in front of the mirror in a systematic way, such as if assessing the relationship between the mirror-image and its own movements [37]. Prior et al. [37] in magpies considered movements of the head or the whole body back and forth and left and right in front of the mirror to be contingent behaviour. In jackdaws, we witnessed head movements to the left and right and/or movements of the whole body to the left and right (Video S5 in Supplementary Material), slowly opening of the beak Video S3 in Supplementary Material) and, very frequently, bristling of the feathers and shaking of the plumage (Video S6 in Supplementary Material). However, these latter two types of behaviour were frequent without the mirror, also, so we did not consider them evidence of self-contingency. On one occasion, the jackdaw Blue-Blue performed a series of peculiar movements with its head and neck that could be considered self-contingent behaviour (see Video S7 in Supplementary Material). Most parameters were scored from the videotapes. The exception was the number of looks behind the mirror while stopping to look back during the first and second experimental stages, a parameter that was scored during direct observation. Two of the authors (M.S. and T.P.C.) independently scored the behaviour of the jackdaws based on the video recordings of 29 bird/sessions (17.4%) randomly chosen and their scores were highly correlated both when considering behavioural variables (*N* = 92, *r_s_* = 0.97, *p*<0.001) and when considering variables related to quantification of time (*N* = 37, *r_s_* = 0.96, *p*<0.001).

Statistical analyses were performed using SPSS 20.0.0, except McNemar tests that were performed in R 3.0.0 [44].

## Results

We found significant differences in the jackdaws' behaviour between stage 1 and stage 2 (i.e. between the 30-min baseline session with a non-reflective cardboard and 150-min mirror exposure sessions) (Table 1). The birds spent more time viewing the mirror than viewing the cardboard and they frequently pecked the mirror, looked behind the mirror and performed contingent behaviour in front of the mirror but not in front of the cardboard (Table 1).

**Table 1:** Jackdaws' behavioural data for experimental stages 1 (30-min baseline session with cardboard) and 2 (150-min mirror exposure sessions).

Subject	Time with view of mirror (secs./h)		Close inspection of (secs./h)		Frequency of pecks to		Frequency of looks behind		Frequency of social behaviours		Frequency of self-contingent behaviour	
	cardboard	mirror	cardboard	mirror	cardboard	mirror	cardboard	mirror	cardboard	mirror	cardboard	mirror
White (F)	1686	2770.8	4	170.4	0	14.8	0	137.2	0	7.6	0	2.4
Green (M)	2158	3042.8	16	702.8	0	5.6	0	10.4	0	4	0	0.4
Orange (M)	3180	2767.6	238	153.2	0	6	0	109.2	0	0.8	0	1.6
Blue-Blue (F)	2880	3581.6	40	269.2	0	6.8	0	0.8	0	0	0	0
Orange-Orange (F)	2164	3220	50	294.8	0	0.8	0	20	0	0.8	0	0.8
Blue (M)	3236	3285.6	360	94.4	0	0.4	0	16.8	0	1.2	0	2.4
Yellow-Yellow (M)	1048	3555.2	4	172.4	0	7.2	20	2	0	2.4	0	0.4
Red (F)	1726	3156.6	18	60.8	0	13.2	0	19.2	0	4.4	0	10.4
Yellow (F)	2510	3200.4	22	790.8	0	14	0	40	0	8.4	0	2.8
Wilcoxon Matched Pairs Test	*T* = 2, *p* = 0.015		*T* = 9, *p* = 0.110		*T* = 0, *p* = 0.008		*T* = 4, *p* = 0.028		*T* = 0, *p* = 0.012		*T* = 0, *p* = 0.012	

F = Female, M = Male. Individuals marked with two rings of the same colour were captured in the wild; individuals marked with only one ring were hand reared in the laboratory. Behavioural data have been standardized to calculate each value per hour. Data corresponding to the mirror column has been calculated as the mean value of the three sessions performed with the mirror. Looks behind the mirror involves going behind the mirror and stopping to have direct visual inspection at close range.

In the third experimental stage (in which jackdaws could choose between two identical compartments in the experimental box, one provided with a mirror and the other with a grey non-reflective cardboard), all subjects other than Blue spent more time in the mirror compartment (Table 2). During the first day, when the entrance to the mirror compartment was closer to the entrance to the experimental box, jackdaws entered the mirror compartment in 19 out of 27 (70.4%) sessions. However, during the second day, when the entrance to the cardboard compartment was closer to the entrance to the experimental box, they entered into the cardboard compartment only in seven out of 27 (25.9%) sessions. Thus, jackdaws showed a clear preference for the mirror compartment independently of which entrance was the closer to the box entrance (Fisher exact test: *p* = 0.002). In fact, seven out of nine jackdaws entered the cardboard compartment at least once, but in all cases rapidly changed to the mirror compartment.

**Table 2:** Jackdaws' behavioural data for experimental stage 3 (choice between mirror and cardboard compartments).

Subject	Time spent in the mirror compartment (sec)	Time spent in the cardboard compartment (sec)	Frequency of close inspection	Frequency of pecks	Frequency of looks behind the mirror	Frequency of social behaviours	Frequency of self-contingent behaviour
White	3501.5	98.5	0/37.5	0/0	0	0/66.5	0/4.5
Green	3570.6	29.5	0/125	0/2.5	1	0/0	0/7.5
Orange	3254.5	345.5	0/118.5	0/23.5	0.5	0/0	0/3.5
Blue-Blue	3570.5	29.5	0/367	0/2	1.5	0/1	0/2.5
Orange-Orange	3567.5	32.5	0/184	0/1	3.5	0/0	0/1
Blue*	425	0	0/3.5	0/0	0	0/0.5	0/2.5
Yellow-Yellow	2965	635	0/92	0/0	0	0/0	0/1
Red	3577.5	22.5	0/243	0/10.5	2	0/0.5	0/9.5
Yellow	3584.5	15.5	0/297.5	0/3.5	2	0/0	0/3

Behavioural data have been standardized to calculate each value per hour. In each case, the first value shows the number of bouts performed in front of the cardboard and the second those performed in front of the mirror. Data has been calculated as the mean value of the six sessions performed in the box separated in two compartments. Looks behind the mirror in these experimental sessions, in which the mirror (or the cardboard) is on the wall, involve going to the edge of the mirror and have a lateral look.

* : The jackdaw Blue spent most of its time in the corridor without entering any of the compartments. For information on sex and status (captured in the wild or hand reared in the laboratory) of each individual see Table 1.

We also quantified (Table 2, Table 3) time spent on close inspection of the mirror-image, number of looks behind the mirror, and instances of social behaviour (see one jackdaw's attack-like behaviour towards the mirror in Video S2 in supplementary material) and of self-contingent behaviour. None of the jackdaws closely inspected the cardboard nor pecked at it, but most of them performed these two types of behaviour with the mirror both during the third (Table 2) and fourth experimental stages (Table 3).

**Table 3:** Jackdaws' behavioural data for experimental stage 4 (mark test).

Subject	Frequency of close inspection	Frequency of pecks	Frequency of looks behind the mirror	Frequency of social behaviours	Frequency of self-contingent behaviour
White	15/121	5.25/1.5	0	0/3.75	0/3
Green	8/191	0/0.75	0	0/0.75	0/8.25
Orange	13.5/150	13.5/3.75	0	0/0	0/0
Blue-Blue	24.75/282.75	11.25/29.25	0	0/0	0/112.5
Orange-Orange	15/77.25	0/0	0	0/0	0/0.75
Blue	6.75/33	0/0	0	0/1.5	0/0
Yellow-Yellow	0/63	0/0	0	0/0	0/0
Red	1.5/222	3/45.75	0	0/0	0/17.75
Yellow	12/324	0/9	0	0/0	0/2.25

Behavioural data have been standardized to calculate each value per hour. In each case, the first value shows the number of bouts performed in front of the cardboard and the second those performed in front of the mirror. Data has been calculated, the first number as the mean of the four sessions performed with the non-reflective cardboard, and the second as the mean of the four sessions performed with the mirror. Looks behind the mirror, as the mirror (or the cardboard) is on the wall, involve going to the edge of the mirror and have a lateral look. For information on sex and status (captured in the wild or hand reared in the laboratory) of each individual see Table 1.

In summary, with respect to mirror preference and mirror exploration, eight out of nine jackdaws spent significantly more time in the mirror compartment and closely inspected their own image in the mirror (Table 1). Social behaviours were significantly more frequent in front of the mirror during the second experimental stage (Table 1). During stage 3 however, no significant differences in social behaviour were found between mirror and cardboard (control) sessions (Wilcoxon matched pairs test: *T* = 0, *N* = 9; *p* = 0.068). Self-contingent behaviour was significantly more frequent in front of the mirror, as all jackdaws showed at least one instance of self-contingent behaviour at least in one of the experimental sessions (Table 1; Stage 3: Wilcoxon matched pairs test, *T* = 0, *N* = 9; *p* = 0.008).

All our jackdaws bristled their feathers and shook their plumage in all stages (Table S1). The frequency of these behaviours was influenced by the presence of the mirror and the sticker. Differences between similar conditions were all significant, except between cardboard with and without sticker (Wilcoxon matched pairs test: cardboard/no sticker vs. cardboard/sticker, *T* = 9, *p* = 0.129; mirror/no sticker vs. mirror/sticker, *T* = 3, *p* = 0.027; cardboard/sticker vs. mirror/sticker, *T* = 0, *p* = 0.008; cardboard/no sticker vs. mirror/no sticker, *T* = 0, *p* = 0.008; *N* = 9 in all tests; p-values were corrected following False Discovery Rate (FDR; [45]) method). In four jackdaws, active feather movement happened three out of eight times (37.5%) during the 30 seconds prior to mark-directed actions under cardboard (control) conditions. In addition, three jackdaws showed active feather movement in two out of nine times (22.2%) during the 30 seconds prior to mark-directed actions under mirror (experimental) conditions.

### Mirror test

None of the jackdaws performed mark-directed actions in both the cardboard/black sticker and the mirror/black sticker conditions. In the mirror/colour condition three of the jackdaws performed mark-directed actions (see Video S8 in supplementary material; Table 4). In the cardboard/colour condition four did so (Table 4). Mark-directed behaviours were performed only under the colour sticker conditions. We found no significant differences in the number of jackdaws that showed mark-directed behaviours with colour and black sticker (Table 4; McNemar test, *p* = 0.423). Moreover, number of jackdaws that showed mark-directed actions did not differ when they were exposed to the mirror or the cardboard (Table 4; McNemar test; *p* = 0.752). It is worth mentioning that jackdaws Blue-Blue and Green performed mark-directed actions more frequently when provided with the colour than when provided with the black sticker (Table 4).

**Table 4:** Jackdaws' frequencies of self-directed behaviours in experimental stage 4 (mark test).

Subject	Cardboard/Colour	Cardboard/Black	Mirror/Colour	Mirror/Black
White	0/0	0/3	0/4	0/0
Green	2+/0	0/0	2/0	0/0
Orange	0/0	0/0	1+/0	0/1
Blue-Blue	8/0	0/0	6/0	0/0
Orange-Orange	0/0	0/0	0/1	0/0
Blue	0/0	0/0	0/0	0/0
Yellow-Yellow	0/0	0/0	0/0	0/0
Red	1/0	0/3	0/0	0/8
Yellow	1+/0	0/0	0/2	0/2

Birds were given a total of eight sessions each, two sessions per experimental condition (in columns). Numbers show the total number of self-directed behaviours in both sessions. In each case, the first value shows the number of mark-directed actions, and the second refers to self-directed actions towards other parts of the body. A plus sign (+) indicates that the bird was successful removing the sticker in any of the sessions. None of the individuals removed the sticker more than once.

During the last stage Blue-Blue did not perform any mark directed action with any of the sticker colours, but performed one self-directed action to another part of its body (touching wing feathers with its beak) during the test with the black sticker.

## Discussion

Throughout stages 1 to 3, jackdaws consistently showed interest for the mirror, spending significantly more time viewing and inspecting it than the cardboard (in stage 1 and in one of the sessions in stage 3) (Tables 1 and 2). Jackdaws frequently looked behind the mirror and mostly chose the compartment with the mirror instead of the compartment with the non-reflective cardboard. In addition, self-contingent behaviour started as soon as the mirror was introduced to jackdaws (i.e. in the first session of stage 2). In contrast, the previous magpie mirror study reported that their subjects behaved as if they were testing contingency only after 150 min of mirror exposure [37]. These results indicate that jackdaws were actively interested in the mirrors, in a similar way to that described for magpies [37], great apes that usually do not pass the mark test [46], and even great apes that usually do pass the test [26], [47].

Thus, our results from stages 1–3 with jackdaws seemed very promising because, as proposed by Heschl & Fuchsbichler [43], passing the mark test possibly requires previous, intensive mirror-image exploration allowing the animal to develop a detailed knowledge of its own appearance. Also, according to Prior et al. [37], individuals that showed a high preference for the mirror later performed mark-directed actions.

However, although our jackdaws presented one of the highest levels of interest for the mirror so far reported [26], [37], [45], [46], they did not exhibit significant differences in mark-directed behaviour across the experimental conditions of stage 4 (i.e. stickers were removed in front of the cardboard as well as in front of mirrors, up to four jackdaws showed this behaviour). In three occasions, the sticker was removed by the bird's neck movements, presumably in the process of attempting to reach the sticker with its beak (see Video S8 in supplementary material). Jackdaws never tried to remove the sticker with their feet as did magpies in the Prior et al. experiment [37].

Corvid black feathers are iridescent, preventing us from producing a black (control) sticker that could remain completely invisible when subjects moved in front of the mirror. However, unlike in the previous magpie study in which subjects performed self-directed behaviour towards the mark area when marked with black stickers [37], our jackdaws made no mark-directed behaviour in neither the cardboard or mirror conditions with a black sticker. This result is most intriguing. There are two plausible explanations for both the jackdaws' lack of mark-directed behaviour when a black sticker was used and their mark-directed behaviour when a red or yellow sticker was used. First, it is possible that jackdaws could visually perceive the red/yellow sticker (at least the birds Blue-Blue and Green) but not the black sticker among their throat feathers. In our study the jackdaws Blue-Blue and Green have shown evidence of perceiving the coloured mark, but they performed a similar number of mark-directed actions with the cardboard and with the mirror. Second, it is also possible that jackdaws were able to visually perceive the black sticker in the mirror, but did not react to its presence because it shared their feathers' colour properties. In contrast, red and yellow stickers could be perceived as threatening or intolerable (birds constantly take care of their feathers and spend a great deal of time tending to them), which would lead to an increase of mark-directed actions and finally to its removal. In either scenario, it is not unconceivable that our subjects were also able to tactilely perceive the stickers (especially when bristling and shaking their feathers; see below for a more detailed discussion of this aspect).

The previously discussed result concerning the black sticker and our results on the mark test can be interpreted as a consequence of chance because the frequency of jackdaw mark-directed actions was similar in absence and in presence of the mirror. This is in agreement with one of the main criticisms made against mirror self-reflection studies, i.e. that an animal could made self-directed actions without using its reflection in the mirror, with its position looking towards the mirror or not being incidental [29], [47], [48].

Our result that the frequency of jackdaw mark-directed actions was similar in absence and in presence of the mirror cast doubts on the conclusion of Prior et al. [37] affirming that magpies are capable of mirror self-recognition. The present findings suggest the possibility that mark-directed actions reported in two out of five magpies [37] could be an artefact of the methodology, i.e. a larger sticker fixed using a double-sided adhesive tape on the sensitive throat feathers could be detected tactilely by the bird, which would try to remove it whether in front of the mirror or not (see below). In the Prior et al. [37] study, their magpies performed mark-directed actions more frequently in front of the mirror, but also in non-mirror conditions. However, in our jackdaws, Green, Orange and Blue-Blue performed mark-directed actions in the mirror/colour condition and two of the three previously mentioned jackdaws (i.e. Green and Blue-Blue) and two additional ones (i.e. Red and Yellow) made mark-directed behaviours in the cardboard/colour condition (Table 4). These results raise the possibility that the sample was too small in Prior et al. [37] study to elicit the same inconsistent mark-directed behaviour showed by jackdaws in the present study.

Both Prior et al. [37] and our study show strong evidence that birds sensed the marks. The key question is what is the most important sense involved in mark detection, vision or touch? Magpies (three cases in [37]) and especially jackdaws (this study) perform mark-directed actions under non-mirror conditions. This strongly suggests that they are somehow able to detect the sticker independently of the vision sense. A likely possibility is that the bird, when moving feathers (e.g. bristling or shaking them), which are mobile and presumably more sensitive than mammalian hair, could detect the sticker and try to remove it. Prior et al. discuss in detail that the sticker cannot be seen directly without using the reflection of the mirror, but they do not discuss this possibility of being detected through feather sensitivity. To bristle the feathers and to shake the plumage are very common and frequent actions in birds, and these movements could favour the detection of the sticker. Our results indicate that the frequency of bristling and shaking in jackdaws is influenced by both the mirror and the sticker, which suggests that the reflection of their image in the mirror increases the frequency of bristling and shaking activities, but also, that a fixed sticker also increases the frequency of these activities regardless of the presence of a mirror or a cardboard, which indicates that jackdaws were able to tactilely perceive the sticker. Methodological issues have often been suggested as a potential source of erroneous results in experimental studies on the mark test [27], [29], [34], [48], and the use of tactilely perceivable self-adhesive stickers could be one of them. Prior et al. [37] did not quantify bristling and shaking behaviour but magpies also made such movements (see e.g. Video S7, the three scenes, and Video S8).

The previously mentioned fact of the iridescence of feathers and considerations described above reveal important methodological issues ignored to date. They represent a source for alternative explanations for previously reported [37] avian mark-directed behaviour and challenge the view that stickers can be used as an appropriate replacement for the paint marks used in mammalian mirror studies. This is especially true for black stickers as being considered a good alternative to sham marking [26], [35], [36], [49], [50].

The methodological problems pointed out by our study on jackdaws (i.e. the difficulty of producing a true sham mark control and the likely possibility that birds might perceive the self-adhesive stickers) when replicating the previous methodology used by Prior et al. [37] encourage us to suggest a more appropriate marking method for future avian mark tests. In birds, the mark should be painted by using a paint which does not agglomerate the feathers or, at least, that would allow perfect separation of feathers when dried, for instance typing correction fluid. Later, when dried, the feathers will be carefully separated. In a control group the mark would be painted with aliphatic hydrocarbons, the solvent of the correction fluid, and subsequently separated as well.

In addition to the methodological problems pointed out by our study on jackdaws, differences between Prior et al. [37] results on magpies and our results on jackdaws could also be affected by differences between the two species. However, this does not seem likely because the two main characteristics that would favour mirror-induced self-directed behaviour showed opposite tendencies in the two species: magpies store food more frequently than do jackdaws [51], but jackdaws present a more complex social behavior than do magpies [43].

The study by Prior et al. [37] has enormous biological and cognitive implications. The fact that magpies are capable of mirror self-recognition, a capacity that has not evolved in most primate species [52] (but see [25]), means that magpies are at the same cognitive level of chimpanzees (*Pan troglodytes*). This implies that mirror self-recognition has evolved independently in the magpie and great apes, which diverged 300 million years ago, and signifies that the neocortex, which is not present in the bird's brains, is not a prerequisite for mirror self-recognition as previously believed [37]. We do not mean to say that our results render the findings reported by Prior et al. [37] artefactual in nature. We only wish to point out that the tremendous biological and cognitive implications drawn from their interpretation of their experimental data call for caution. In our view, in order to unambiguously demonstrate that magpies are able to consistently pass the mark test, their experiment needs to be replicated (with a larger sample) using improved methodology that accounts for the alternative explanations and the potential confounding issues revealed by our jackdaw study.

## Supporting Information

Table S1 **Frequency of feather bristling and shaking by jackdaws in different experimental stages (number of behaviours per hour).** In stages 1and 2, jackdaws were tested without stickers. In stage 4, jackdaws were tested with either black or coloured stickers.
(DOC)

Video S1 **A jackdaw is released at the entrance of the corridor for the experimental stage 3 (choice between mirror and cardboard compartments).**
(MP4)

Video S2 **Two instances of aggressive attack-like behaviour towards the mirror.**
(MP4)

Video S3 **Other potential self-contingent behaviour: slowly opening of the beak.** In no case did we observe this behaviour in any social interaction or in front of the cardboard.
(MP4)

Video S4 **An instance of close inspection of mirror.**
(MP4)

Video S5 **Self-contingent behaviour: movements of the whole body left and right in front of the mirror.** In no case did we observe this behaviour in any social interaction or in front of the cardboard.
(MP4)

Video S6 **An example of a jackdaw first bristling its feathers and later shaking its plumage.** Both types of behaviour were frequent in the cardboard conditions also, so we did not consider these actions evidence of self-contingency.
(MP4)

Video S7 **Rare movements performed with its head and neck by Blue-Blue in direct contact with the mirror, which could be considered self-contingent behaviour.**
(MP4)

Video S8 **Mark-directed actions: attempts to reach the sticker with the beak.**
(MP4)
