# Supplementary material for: Mirror-Mark Tests Performed on Jackdaws Reveal Potential Methodological Problems in the Use of Stickers in Avian Mark-Test Studies
Source: PLoS One. 2014 Jan 27;9(1):e86193. doi: 10.1371/journal.pone.0086193 (PMC3903501; doi:10.1371/journal.pone.0086193)
Supplement: Table S1 — Frequency of feather bristling and shaking by jackdaws in different experimental stages (number of behaviours per hour). In stages 1and 2, jackdaws were tested without stickers. In stage 4, jackdaws were tested with either black or coloured stickers. (DOC) [file pone.0086193.s001.doc]

**Table S1**. Frequency of feather bristling and shaking by jackdaws at different experimental stages (number of these behaviours per hour). In stage 1and 2, jackdaws confront the experiments without any sticker, since in stage 4 jackdaws were wearing stickers (sham or colour ones).

| Jackdaw | Stage 1  (cardboard) | Stage 2  (mirror) | Stage 4  (cardboard) | Stage 4  (mirror) |
| --- | --- | --- | --- | --- |
| Green | 0.00 | 15.20 | 5.25 | 22.50 |
| Yellow | 0.00 | 12.00 | 2.25 | 33.00 |
| White | 1.50 | 18.00 | 15.00 | 24.00 |
| Orange | 0.50 | 14.40 | 17.25 | 25.50 |
| Yellow-yellow | 0.50 | 5.20 | 0.00 | 3.75 |
| Red | 3.00 | 20.00 | 11.25 | 31.50 |
| Blue | 3.00 | 17.60 | 5.25 | 14.25 |
| Blue-Blue | 1.00 | 7.20 | 16.50 | 43.50 |
| Orange-orange | 2.00 | 20.40 | 12.75 | 35.25 |
| Average | 1.30 | 14.40 | 9.50 | 25.90 |
| Standard deviation | 1.2 | 5.4 | 6.4 | 11.8 |
